# FOXO family isoforms

**DOI:** 10.1038/s41419-023-06177-1

**Published:** 2023-10-27

**Authors:** Bruno F. Santos, Inês Grenho, Paulo J. Martel, Bibiana I. Ferreira, Wolfgang Link

**Affiliations:** 1 Algarve Biomedical Center Research Institute-ABC-RI, University of Algarve, Campus de Gambelas, 8005-139 Faro, Portugal; 2 Algarve Biomedical Center (ABC), University of Algarve, Campus de Gambelas, 8005-139 Faro, Portugal; 3 Faculty of Medicine and Biomedical Sciences, University of Algarve, Campus de Gambelas, 8005-139 Faro, Portugal; 4 Centro Hospitalar Universitário do Algarve (CHUA). Rua Leão Penedo, 8000-386 Faro, Portugal; 5 Center for Health Technology and Services Research (CINTESIS)@RISE, University of Algarve, Campus de Gambelas, 8005-139 Faro, Portugal; 6 Instituto de Investigaciones Biomédicas “Alberto Sols” (CSIC-UAM). Arturo Duperier 4, 28029 Madrid, Spain

**Keywords:** Transcription, Oncogenesis

## Abstract

FOXO family of proteins are transcription factors involved in many physiological and pathological processes including cellular homeostasis, stem cell maintenance, cancer, metabolic, and cardiovascular diseases. Genetic evidence has been accumulating to suggest a prominent role of FOXOs in lifespan regulation in animal systems from hydra, C elegans, Drosophila, and mice. Together with the observation that FOXO3 is the second most replicated gene associated with extreme human longevity suggests that pharmacological targeting of FOXO proteins can be a promising approach to treat cancer and other age-related diseases and extend life and health span. However, due to the broad range of cellular functions of the FOXO family members FOXO1, 3, 4, and 6, isoform-specific targeting of FOXOs might lead to greater benefits and cause fewer side effects. Therefore, a deeper understanding of the common and specific features of these proteins as well as their redundant and specific functions in our cells represents the basis of specific targeting strategies. In this review, we provide an overview of the evolution, structure, function, and disease-relevance of each of the FOXO family members.

## Facts

- The four isoforms of FOXO transcription factors in mammals have both overlapping and non-redundant functions.
- Isoform-specific, non-redundant functions are regulated by intrinsic and context-dependent mechanisms including differences in the paralog genes, transcripts, and proteins, their regulation by miRNAs, post-translational modification (PTMs), and binding partners as well as their cell-specific expression and regulation.
- FOXO isoforms exert a differential role in human aging and in several age-related diseases.
- Pharmacological inhibition or activation of FOXO proteins for therapeutic or preventive purposes has become feasible.

## Open questions

- What is the contribution of tissue-specific expression and co-expression patterns versus isoform-specific upstream regulation and downstream signaling to non-overlapping functionalities of the FOXO isoforms?
- When and why do FOXO isoforms exert redundant functions under physiological and pathological conditions?
- Is isoform-specific manipulation of FOXO activity feasible and beneficial?
- Which are the clinical indications best suited for isoform-specific FOXO targeting?

## Introduction

FOXO proteins represent a sub-family of the forkhead box family (FOX) superfamily of proteins. Originally identified in the fruit fly, the FOX proteins can be found in virtually all eukaryotes, from yeast to vertebrates, but not in plants [1–4]. The FOX superfamily is one of the largest transcription factor families in humans. It comprises 19 sub-families of tissue specific transcription factors that contain a highly conserved DNA-binding domain of approximately 100 amino acids, referred to as the forkhead box domain. Based on sequence similarities in particular in the regions outside the forkhead box domain, the sub-families have been classified from FOXA to FOXR with at least 41 genes currently identified in humans [5]. Members of the FOX protein family play important roles in a wide range of physiological and pathological processes including FOXE3 which has been shown to be involved in eye development [6], mutations in FOXP2 can cause speech and language disorders [7], mutations in FOXA1 have been linked to several cancers [8–10] and FOXM1 has emerged as a promising therapeutic target in cancer [10]. The proteins of the FOXO sub-family are evolutionarily well conserved in metazoans. While Hydra vulgaris, Caenorhabditis elegans, and Drosophila melanogaster express a unique FOXO factor (FOXO, daf-16, and dFOXO, respectively), in humans, the FOXO subfamily is composed of four members: FOXO1, FOXO3, FOXO4, and FOXO6. Through their forkhead box domains FOXO transcription factors can specifically recognize two different DNA-response elements, the DAF-16 binding element (DBE) 5´-GTAAACAA-3´ and the insulin-responsive element (IRE) 5´-(C/A)(A/C)AAA(C/T)AA-3´, whose core sequence is 5´-(A/C)AA(C/T)A-3´ [11–15]. It is important to stress that FOXO3 is capable of partially replacing C elegans FOXO/daf-16, proving the orthology [16]. Furthermore, transcription-independent functions of FOXO proteins have emerged including the interaction between ATM and FOXO3 to regulate DNA damage responses [17], FOXO3-mediated recruitment of p53 to the cytoplasm promoting apoptosis [18], and ATG7 binding to cytosolic, acetylated FOXO1 influencing autophagy [19]. It is important to note that whereas FOXO proteins have emerged as potential targets for the therapeutic development of drugs and geroprotectors [20], isoform-specific targeting might increase their specificity and benefit.

### Definitions and nomenclature

**Fig. 1:**
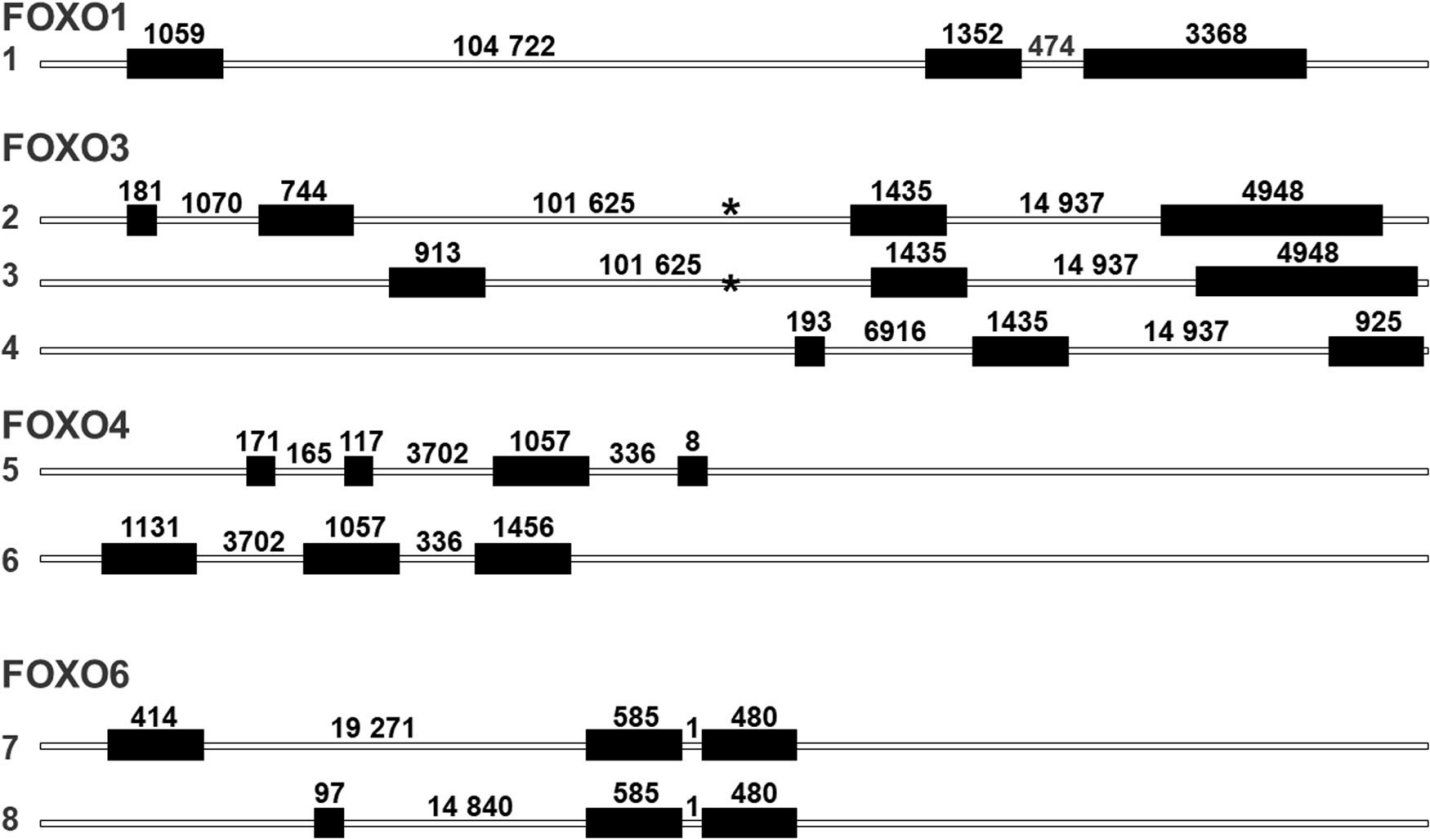
Intron-exon structures of the protein coding FOXO variants. (1) ENST00000379561.6 (FOXO1-201); (2) ENST00000343882.10 (FOXO3-201); (3) ENST00000406360.2 (FOXO3-202); (4) ENST00000540898.1 (FOXO3-203); (5) ENST00000341558.3 (FOXO4-201); (6) ENST00000374259.8 (FOXO4-202); (7) ENST00000641094.2 (FOXO6-202); (8) ENST00000686812.1 (FOXO6-203). Black rectangles represent exons, white bars represent 5′ upstream sequences, introns, and 3′ downstream sequences. Asterisks indicate the position of the rs2802292 polymorphism in FOXO3. Numbers above the black rectangles indicate the size (bp) of the corresponding exons, and numbers above the white bars indicate the size (bp) of the corresponding introns. (www.ensembl.org).

The term isoform has been ambiguously used in the literature as it can refer to different concepts. Isoforms can describe different proteins produced by a single gene or similar proteins derived from different genes. A single gene can give rise to several distinct proteins by using alternative promoters or through alternative splicing. Conversely, paralogous genes generated by duplication of a single original gene can produce very similar proteins. Indeed, the unique FOXO genes in *C elegans* and in *Drosophila*, daf-16 and dFOXO, respectively give rise to various isoforms, while the FOXO paralogs FOXO1, 3, 4, and 6 in mammals produce similar proteins. FOXO2 is a reported synonym for FOXO3, and FOXO5 is the FOXO3 ortholog in fish. FOXO isoforms in humans are generated through both mechanisms. In this review article, the term isoforms refer to the paralog-derived isoforms while the term variants will be used for the isoforms derived from single FOXO genes. The paralogous FOXO genes encode for the FOXO isoforms FOXO1, 3, 4, and 6, here referred to as paralog-derived isoforms, and each of these single genes can generate variants of these proteins. Human FOXO proteins are encoded by four different genes and one pseudogene located on different human chromosomes. These FOXO paralogous genes account for the diversity of functions of FOXO family proteins. While single copy genes suffer from strong negative selective pressure to evolve conservatively, gene duplications can generate redundant copies of genes relieving the selection pressure on one of them and opening up the possibility to adopt new functions. Wang et al. suggested that two major duplications of FOXO genes had occurred early in the vertebrate lineages. The first duplication leading to the emergence of two lineages which evolved into FOXO3/6 and FOXO1/4, and the second duplication finally resulted in FOXO6 and FOXO3, and FOXO1 and FOXO4 [21]. Whereas FOXO3 represents the closest ortholog of C elegans daf-16 and Drosophila´s dFOXO, FOXO6 is the most distant and fast-evolving member of the FOXO family with a large number of accumulated structural changes [21, 22]. Indeed, FOXO6 lacks several regulatory mechanisms shared by the rest of the paralog-derived isoforms as discussed below [23]. It has been suggested that FOXO3 has retained most of the ancestral functions and that FOXO1 and FOXO4 are redundant and may have acquired additional functions [24]. Historically, FOXO1, 3, and 4 have been named FKHR, FKHRL1, and AFX, respectively, and FOXO3 was named FOXO3a because of the existence of the presumed pseudogene FOXO3b. A recent study reveals that the FOXO3b locus encodes a protein without DNA-binding activity constitutively localized in the cytosol [25]. The different human FOXO genes also give rise to different FOXO variants. FOXO1, FOXO3, FOXO4, and FOXO6 are located on human chromosomes 13, 6, X, and 1 respectively. FOXO1 comprises 3 exons spanning a genomic distance of 140 kb. FOXO3 contains 4 exons and 3 introns, but only the largest exons 2 and 3 with 1.4 kb and 4.9 kb encode the protein (Fig. 1). FOXO4 comprises 3 exons, while the genomic structure of FOXO6 has not been reported. Murine FOXO6 contains two putative exons divided by an 18 kb long intron [22]. FOXO3b is located on human chromosome 17 and contains exons 2–4 of FOXO3. Table 1 summarizes the characteristics of FOXO paralog-derived isoforms.

**Table 1:** FOXO paralog-derived isoforms.

Gene	Aliases	Exons	Chromosome	Number of variants	Amino Acids	Mol. Weight (kDa)
FOXO1	FKHR	3	13	4	655	70
FOXO3	FKHRL1	4	6	3	673	72
FOXO3b	FKHRL1P1, ZNF286B	3	17	4	873	290
FOXO4	AFX	3	X	4	505	54
FOXO6		3	1	7	492	61

### Single FOXO gene-derived isoforms

While several paralog-derived variants have been reported for each FOXO isoform (Table 2), none of them has been thoroughly characterized. Through differential upstream exon usage, the FOXO3 gene gives rise to three different proteins. A recent study found the two full-length variants present in almost all FOXO3-expressing tissues, while the expression of the truncated isoform is more restricted [26]. The full-length and the truncated variants exhibit differences in their structures. The truncated isoform lacks important parts of the FOXO3 protein, and it is unlikely that it acts as a transcription factor to regulate FOXO3 target genes. Intriguingly, these variants display a considerable tissue specificity and an association of the longevity allele rs13217795 with increased levels of full-length FOXO3 isoforms transcript in peripheral blood and a decrease in truncated FOXO3 isoforms in skeletal muscle has been reported [26]. These data may suggest that the longevity effect of single nucleotide polymorphisms (SNPs) at the FOXO3 locus may in part derive from a shift in isoform usage. Interestingly, in *C. elegans*, two identified isoforms, daf-16a and daf-16 d/f display differential tissue enrichment, preferential modulation by upstream kinases, and regulate distinct and overlapping target genes, but functionally cooperate to fine-tune insulin/IGF-1 signaling and lifespan of the worm [27]. Another recent study identified FOXO3A-Short (FOXO3A-S), a primate-specific FOXO3A transcriptional isoform encoding a major longevity-associated SNP within its 5′-untranslated region [28]. The longevity T-allele of rs9400239 in FOXO3A-S is associated with higher insulin-stimulated peripheral glucose clearance rates. The two FOXO4-derived variants ENST00000341558.3 which is ubiquitously expressed and ENST00000374259.8 lacking amino acids 58–112 and expressed predominantly in the liver, kidney, pancreas, heart, and placenta [27, 29] have been identified. In addition, the presence of three shorter FOXO4-derived variants was detected in human cancer cell lines [30]. Two shorter N-terminal FOXO4 proteins containing 90 and 101 amino acids are produced by alternative splicing and a protein lacking the first 197 amino acids at the N-terminus result from the usage of a downstream start site. While the N-terminal proteins were unstable, both the ENST00000374259.8 and the N-terminal deletion variant of FOXO4 lost the ability to transactivate the FOXO4 target gene, BCL6 in a dominant-negative manner. These variants also fail to suppress cyclin D2 gene expression and do not induce cancer cell death suggesting a possible oncogenic effect. It is important to note that the functional significance of most of the paralog-derived variants remains to be established.

**Table 2:** Variants derived from single FOXO genes.

Gene	Variant version	Type	Exons/Coding	Length	Amino acids
FOXO1	ENST00000655267.1	Processed transcript	3/0	509	0
	ENST00000660760.1	Processed transcript	3/0	491	0
	ENST00000379561.6	Protein coding	3/2	5779	655
	ENST00000473775.1	Processed transcript	3/0	178	0
FOXO3	ENST00000540898.1	Protein coding	3/1	2553	453
	ENST00000343882.10	Protein coding	4/2	7308	673
	ENST00000406360.2	Protein coding	3/2	7296	673
FOXO4	ENST00000341558.3	Protein coding	4/4	1353	450
	ENST00000374259.8	Protein coding	3/3	3644	505
	ENST00000464598.1	Processed transcript	2/0	306	0
	ENST00000466874.1	Processed transcript	3/0	657	0
FOXO6	ENST00000686812.1	Protein coding	3/3	1162	364
	ENST00000641094.2	Protein coding	3/3	1479	492
	ENST00000372591.1	Processed transcript	2/0	2398	0
	ENST00000630406.6	Protein coding	3/3	1476	492
	ENST00000642843.2	Protein coding	2/2	3,086	559
	ENST00000643181.1	Protein coding	3/3	1476	492
	ENST00000643531.2	Protein coding	2/2	3086	559

### Possible source of isoform-specific functions

**Fig. 2:**
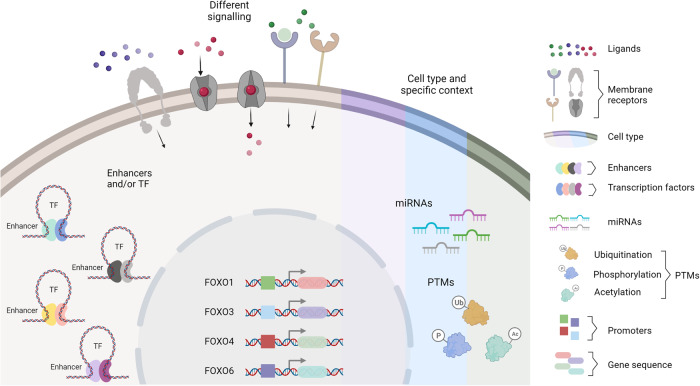
The interplay between intrinsic factors and context-dependent mechanisms determines the redundant and isoform specific functions of FOXOs. FOXO isoforms can be differentially regulated by different mechanisms which include intrinsic characteristics (promoters and gene sequences) of the genes encoding for FOXO1, 3, 4, and 6 or by cell type-specific signaling events or the specific expression of transcription factors, miRNAs, or enzymes that mediate PTMs of FOXOs.

As discussed above, during vertebrate evolution, four paralog-derived FOXO isoforms emerged from a single gene in invertebrates. A large body of evidence has indicated that FOXO1, 3, 4, and 6 have redundant and isoform-specific functions. Most remarkable, full body knockout of Foxo1 in mice leads to defects in vasculature and is embryonic lethal [31]. Whereas Foxo3 knockout mice display oocyte exhaustion and are sterile, FOXO4 and FOXO6 knockout mice only present mild phenotypes. Intriguingly, the simultaneous conditional deletion of Foxo1, 3, and 4 results in a cancer-prone phenotype [32]. Invertebrate FOXOs have been shown to play an important role in invertebrate longevity including *Hydra, C elegant*, and *drosophila* [33–36]. In mammals, including humans, only FOXO3 has consistently been implicated in longevity. As all paralog-derived FOXO isoforms bind to the same DNA consensus sequences, these isoform-specific effects cannot be mediated by the presence of specific regulatory elements in certain FOXO target genes. The source of isoform specific functions of FOXO proteins are poorly understood. We classify sources of isoform-specific functions into intrinsic and context-dependent mechanisms (Fig. 2). Intrinsic mechanisms refer to the different structural features of the isoforms at the gene, transcript, or protein level, while context-dependent mechanisms describe specific functions mediated by the cellular context such as cell-specific regulation of FOXO isoforms expression, cell-specific binding partners and cell-specific post-translational modifications (PTMs). It is important to note that intrinsic and context-dependent factors can interact and promote an isoform-specific net effect. Differences within FOXO proteins, like the presence or absence of specific amino acids can determine the pattern of PTMs and therefore alter the affinity to different binding partners, defining the subset of FOXO target genes regulated by the isoform. A recent study revealed structural differences between the binding of FOXO1 and FOXO3 phosphorylated at S256 or S253, respectively to 14-3-3 chaperone proteins, providing evidence for isoform-specific binding of FOXO proteins to 14-3-3 [37].

Although it has been shown that all FOXO isoforms bind to the same consensus sequences, subtle differences in the environment of the enhancer and promoter elements might still influence the preferential binding of specific FOXO isoforms to target genes. In agreement with this hypothesis, it has been reported that non-canonical FOXO1 recognition sites, incomplete FOXO1 occupancies at the available insulin response elements, and FOXO1 dimeric interactions may play a role in stabilizing DNA looping [38]. While FOXO factors are known to usually bind as monomers to their binding elements, homo, and heterodimerization of FOXO factors might occur and play a role in isoform-specific regulation of target genes. The presence of isoform-specific regulatory regions in the 3′-untranslated region within the FOXO transcripts can induce mRNA degradation and translational repression of isoforms. On the other hand, isoform-specific expression might be driven by both intrinsic and context-dependent mechanisms. The expression of one FOXO isoform can be due to cis-regulatory elements present in one isoform but absent in the others but can also be influenced by the genomic landscape in a given cellular context determining the accessibility to enhancers and promoters, regulating the expression of FOXO isoform. As the genes encoding the human FOXO proteins lie on different chromosomes the enhancer landscapes are independent from each other facilitating individual expression kinetics. Likewise, the presence of binding partners in a specific cellular context might influence the spectrum of target genes regulated by a FOXO isoform. In order to elaborate on the concept of intrinsic and context-dependent sources of isoform-specific FOXO functions, we will discuss intrinsic differences in the structure of FOXO isoforms, their different regulation by PTMs and miRNAs as well as differences in their expression in the following chapters.

### Structure of FOXO isoforms

**Fig. 3:**
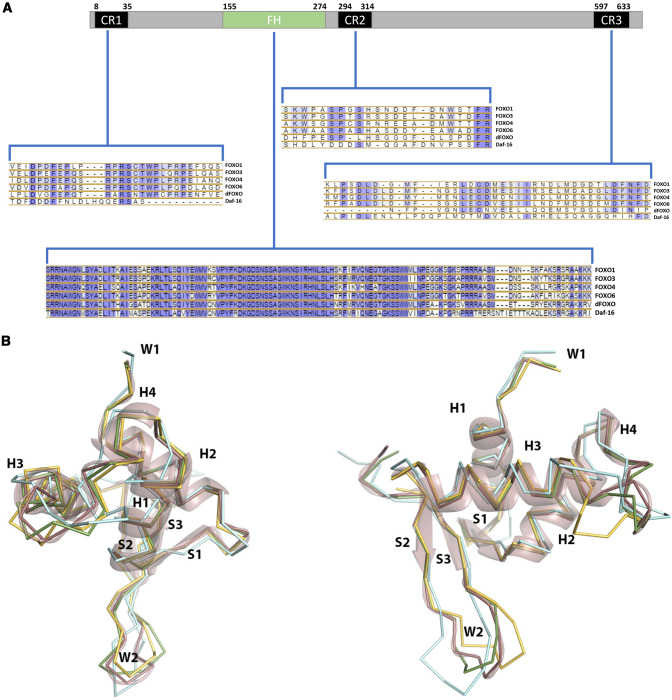
FOXO sequence alignments and structures. **A** FOXO conserved regions with sequence alignments. CR1, CR2, CR3 - conserved regions. FH - forked DNA-binding domain. Sequence numbers for star and end points are for FOXO1. dFOXO - FOXO from Drosophila melaganoster, daf-16 - FOXO homologue from *C. elegans*. **B** Comparison of the structures of FOXO1 (PDB 3c06, pink), FOXO3 (PDB 2uzk, cyan), FOXO4 (PDB 3l2c, yellow), and FOXO6 (Alpha Fold 2 model, green). The FOXO1 structure is also represented as a transparent cartoon. Helices (H), sheets (S), and wing loops (W) are indicated (wing loop 1 is highly disordered in crystallographic structures and only the first residues are visible).

FOXO proteins have a structured winged-helix type of DNA binding domain (DBD), followed by a largely unstructured, flexible “tail” containing nuclear export sequences (NESs) and a transactivation domain (TAD). The forkhead, or “winged helix” DBD (WHD), is the trademark of the FOX protein family [39, 40] and is part of the broader structural class of helix-turn-helix (HTH) DBDs, one of the major motifs of protein-DNA recognition [41]. WHD domains present, however, a few distinctive features setting them apart from other HTH-like domains: the presence of two loops, or “wings”, flanking the recognition helix of the HTH motif, and a longer connecting sequence between helices H2 and H3 [39]. In Fig. 3, the FOXO1 WHD structure illustrates the main architectural features of the WHD. After a short N-terminal sequence, secondary structure elements in the sequence H1-S1-H2-H3-S2-W1-S3-W2 (S:sheet,H:helix, W:wing loop) fold in 3D space to create a compact core where helix H3 (the recognition helix) is supported by interactions with helices H2 and H3. In the FOXO sub-family (and also in other FOX family members), the highly flexible H2-H3 connecting sequence assumes a partially disordered α-helix or 3_10_ -helix (a much less common secondary structure helical conformation) conformation, which may play a role in DNA recognition via nonspecific interactions (helix H4) [42]. Another distinctive feature of the FOXO sub-family is the 5-residue insertion with sequence GDSNS that partially overlaps helix H4 (Fig. 3).

This insertion creates a longer connecting sequence between helices 3 and 4, which appears to be highly flexible, as revealed by both structural studies [42] and molecular dynamics simulations [14]. While flexible, the W1 loop has a much more stable conformation than the W2 loop, the latter frequently displaying an extended or fully disordered conformation not clearly observable in structural experiments. In spite of their disorderly nature, W2 (and to a lesser extent W1) contribute to stabilization FOXO-DNA interactions in an isoform-specific way, and W2 has been shown to be essential for FOXO1-DNA binding. Notwithstanding the high degree of sequence conservation among DBD regions of the FOXO isoforms, significant structural and dynamic differences have been noted, which may have an impact on the specificity of DNA binding [14]. The FOXO1 DBD seems to be more compact and FOXO4-DBD more expanded, with changes in the core structure of hydrophobic residues at the interfaces of helices H1-H3. Flexibility analysis of the NMR structures of the 3 domains also revealed significant differences, FOXO4 being the more flexible DBD with increased conformational heterogeneity. The observed differences are likely to result from the small sequence divergence, and they may impact not only DNA binding but also the interaction with other binding partners like p53.

In contrast to the high level of sequence conservation observed in the DBD, most of the remaining sequence of FOXOs is largely divergent with sequence identity as low as 20%, except for three short-conserved regions CR1, CR2, and CR3 (Fig. 3 and Supplementary Fig. S1). The divergent regions present themselves largely in unstructured form, at least when free from binding partners [43]. With over 70% of their content in disordered form, FOXOs can be classified as intrinsically disordered proteins (IDPs) [44]. This is a common feature among proteins that play important regulatory roles in cellular signaling and control [45]. Also, the TAD domains map entirely within the IDR region, which may enable a large spectrum of co-activators and help to explain isoform-specific functions. As with other IDPs, FOXO disordered regions may adopt ordered conformations upon binding of cognate partners: a fusion peptide of regions CR2C and CR3 of FOXO3 has been shown to adopt helical conformation when bound to the CREB-binding protein (CBP) [46], while these regions are likely disordered when unbound. The experimental NMR structures show two different modes of binding of CR2C-CR3 to CBP, with either the CR2C or CR3 regions structured, highlighting the promiscuous molecular interactions enabled by disordered regions. In FOXO3, the flexibility of the region between the DBD and the CR3 seems to be crucial for interaction with p53, with FOXO3 “clasping” p53 by means of interaction with both the DBD and CR3 [42]. Sequence variability in this region may thus result in different affinities for p53 among FOXO isoforms. In FOXO4, autoinhibition via binding of the CR3 region to the DBD has been demonstrated, with experimental evidence supporting an alpha-helical conformation for CR3 upon binding [47]. In FOXO6, the absence of the nuclear export signal region of CR2 in FOXO6 will likely impede translocation to the cytoplasm, in line with FOXO6 being the more divergent of the 4 isoforms [47]. The unstructured FOXO regions may enable promiscuous binding modes, expanding the spectrum of possible binding partners [43]. Different PTMs in the unstructured region of FOXOs appear to modulate their affinity for binding partners and have been described as the “FOXO code” [48]. In conclusion, sequence, and structural variability among the four FOXO isoforms will impact different functional aspects: while the small sequence variation in the DBD domain leads to subtle variations in dynamics and conformation of DNA binding, the high degree of sequence divergence in the IDRs will likely offer multiple mechanisms of isoform specificity thank different binding partners and affinities, in a largely unexplored landscape.

### Regulation of FOXO isoforms

The main negative and positive regulatory inputs that control the activity of FOXO proteins come from the growth factor-dependent PI3K/AKT pathway and cellular stress signaling, respectively. FOXOs are primarily regulated through a complex combination of PTMs, such as phosphorylation, acetylation, mono and polyubiquitination, methylation, glycosylation, and nitrosylation. Table 3 shows the profile of PTMs for each FOXO isoform. These PTMs can alter FOXO conformation creating specific binding motifs for protein partners, and in turn activate or inhibit their activity [3, 49].

**Table 3:** Post-Translational Modifications of the FOXO Isoforms FOXO1, 3, 4, and 6 by different enzymes that covalently modify these proteins at the listed amino acid residues [141, 142].

Enzyme	Name	FOXO1	FOXO3	FOXO4	FOXO6
Kinase	Akt1	T24, S256, S319	T32, S253, S315	T32, S197, S262	T26, S184
	AKT2		T32, S253, S315	T32, S197	
	AKT3		T32, S253, S315		
	AMPKA1	S22, T649	T179, S399, S413, S555, S588, S626		
	AMPKA2		T179, S399, S413, S555, S588, S626		
	CDK1	S249			
	CDK2	S249, S298			
	CDK4	S249			
	CDK5	S249			
	CK1A	S322, S325	S318, S321	S265, S268	
	DYRK1A	S329	S330		
	ERK1		S12, S294, S344, S425		
	ERK2	S246, S413, S418, S429, S470, T478, T560	S12, S294, S344, S425		
	IKKA		S644		
	IKKB		S644		
	IKKE		S644		
	JNK1		S574	T227, S230, T451, T455	
	JNK2			T451, T455	
	MST1	S212, S218, S234, S235	T179, S209, S215, S231, S232, S243		
	NLK	S329			
	P38A	S416, S432, S470, T478, T560	S7, S12, S294, S344, S425		
	Pim1	T24, S256, S319	T32, S253		
	PKACA	S153, S276			
	SGK1		T32, S253, S315		
	SGK2		T32, S253		
	SGK3		S315		
Phosphatase	PPP2CA		T32, S253		
Methyltransferase	PRMT1	R251, R253			
Acetyltransferase	p300	K245, K248, K262, K265		K186, K189, K407	
Deacetylase	HDAC1			K186, K189, K407	
	SIRT1		K242, K259, K290, K569	K189	
	SIRT2	K245, K248, K262, K265	K259, K290, K569		
	SIRT5		K271, K290		
	SIRT7		K242, K259, K290, K569		

In the presence of insulin, and many other growth factors, the canonical regulator AKT phosphorylates FOXO proteins in the nucleus, at three conserved RxRxxS/T residues. AKT phosphorylates FOXO1 at Thr24, Ser256, and Ser319, FOXO3 at Thr32, Ser253 and Ser315 and FOXO4 at Thr28, Ser193 and Ser258. These covalent modifications create a docking site for the chaperon protein 14–3–3 enabling the CRM1-mediated nuclear export of FOXO factors [50] FOXO nuclear export and preventing the regulation of target genes [3, 51]. As shown in Table 3 several additional kinases have been shown to phosphorylate FOXO isoforms at different sites. It is important to note that some kinases, known to be involved in stress signaling, including JNK, MST1, and AMPK can stimulate the activity of FOXO proteins. FOXO6 only has two conserved AKT phosphorylation sites, Thr26 and Ser184 lacking the conserved C-terminal motif. In addition, the specific CK1 and DYRK1A -dependent kinase consensus sites of phosphorylation are also absent from FOXO6. Although FOXO6 phosphorylation by AKT exerts an inhibitory effect on its activity, it does not result in its nuclear export [52]. Growth factors can also regulate FOXOs at the transcription level since growth factor deprivation can reduce FOXO1, FOXO3, and FOXO4 mRNA levels [53]. Depending on the intensity of the stimuli, FOXO1, FOXO3, and FOXO4 can be temporarily sequestered in the cytoplasm and reactivated without the requirement of de novo protein synthesis, or be ubiquitinated and degraded by the proteasome upon potent or chronic insulin signaling [49].

On the other hand, elevated levels of reactive oxygen species (ROS), nutrient deprivation, or DNA damage, can activate FOXOs to restore cellular homeostasis [3, 49, 51]. Thereby, different stimuli result in different PTMs that collectively help fine-tune the amount of nuclear FOXOs, hence regulating their transcriptional activity. A recent study elegantly shows that the dynamics of FOXO1 and FOXO3 nuclear shuttling are stimulus-dependent [54]. While oxidative stress through H2O2 treatment leads to an all-or-none response and cell death in a dose dependent manner, serum starvation causes low-amplitude pulses of nuclear FOXO and predominantly results in cell-cycle arrest suggesting that the dynamics of FOXO nuclear shuttling dictates different cellular outcomes. Cysteine residues play an important role in redox signaling by reversibly altering protein structure and function. FOXO1 contains seven cysteines, FOXO3 and FOXO4 five, and FOXO6 only four. Only two of these residues are conserved among all four FOXO isoforms. CDK4 kinase and the transcriptional activator p300 can bind to the same conserved cysteines but isoform-specific cysteines interact with different proteins. Interestingly, Putker et al. provided evidence for an isoform-specific regulation of human FOXO3 and FOXO4 by redox signaling. Through isoform-specific cysteines, FOXO3 forms a disulfide-dependent heterodimer with the nuclear import receptors importin-7 (IPO7) and importin-8 (IPO8) which is required for its ROS-induced nuclear translocation [55]. Conversely, disulfide-dependent binding of FOXO4 to the nuclear import receptor transportin-1 (TNPO1) is required for nuclear localization and the activation of FOXO4 [56].

Upon low energy levels, AMPK can phosphorylate FOXO3 in six different residues (T179, S399, S413, S555, S588, and S626) to induce FOXO activity. Only FOXO3 and FOXO4 share the motif flanking Ser413 while the Ser588 motif is solely conserved between FOXO3 and FOXO1 [57]. Although AMPK can phosphorylate other FOXO isoforms, these sites are not fully conserved, and hence, FOXO3 seems to be the preferential target of AMPK. The activity of FOXO proteins can also be regulated by the addition of methyl groups to the side-chains of arginine and lysine residues. Protein arginine methyltransferase 1 (PRMT1) methylates FOXO1 at Arg248 and Arg250, leading to its activation. PRMT1 can also interact with FOXO3 but is unable to methylate it. However, in the absence of PRMT1, PRMT6 specifically methylates FOXO3 at Arg188 and Arg249 leading to its activation. Conversely, PRMT6 interacts with FOXO1 but fails to methylate. Out of the three potential methylation sites in FOXO3, only one is conserved in FOXO1, which might explain why arginines are selectively modified by different PRMTs [58]. Two acetylated lysine sites are highly conserved between all FOXOs isoforms, one site is less conserved and the rest being isoform-specific which may contribute to isoform-specific regulation [58]. Nonetheless, even conserved sites can be regulated by different enzymes. Conserved Lys273 in FOXO1 is methylated by G9a whereas Lys270/271 in FOXO3 is methylated by Set9 [58].

FOXOs activity significantly depends on the interaction with binding partners to regulate transcription. This co-regulation can even occur independently of direct binding of FOXO to DNA either through transcriptional synergy, recruitment of conventional cofactors, proteolytic degradation, transcription factor sequestration, or displacement of regulatory cofactors [59]. These interactions will depend on the availability of binding partners but can also be isoform-specific. Although all the isoforms exhibit an LxxLL motif located at the C-terminal of the Forkhead DNA-binding domain, the surrounding regions diverge among them and this motif is not present in dFOXO or in daf16 in Drosophila melanogaster and *C. elegans*, respectively. As the LxxLL motif in the human FOXO isoforms has been suggested to mediate their interaction with nuclear hormone receptors (NHRs), the isoform-specific differences of the LxxLL motif might play a role in the selective interaction between FOXOs and several NHRs [59].

FOXOs activity can also be regulated at the post-transcriptional level. Table 4 shows several miRNAs capable of regulating different isoforms. By binding to 3′-UTRs of target transcripts, miRNAs can promote RNA degradation or inhibit translation and have been shown to be deregulated in various human diseases [60, 61]. As sequence identity of regions outside the DBD is low between the FOXO isoforms, it is not surprising that there is little overlap between miRNAs identified to be involved in their regulation. Indeed, only hsa-miR-146a-5p targets the four FOXO isoforms. Furthermore, depending on the cellular context, the same miRNA can contribute to different functional outcomes. miR-27a, by targeting FOXO3, can either promote proliferation and invasion of glioblastoma cells [62], or suppress neuronal autophagy after traumatic brain injury [63]. Post-transcriptional regulation can also occur through RNA-binding proteins. In FOXO1 RNA, HuR-binding sites are present in the 3′-UTR and intronic regions, while in FOXO3 these sites are limited to intronic regions. HuR is involved in RNA processing, regulation, and stability. Upon cellular stress, HuR stabilizes FOXO1 mRNA leading to higher protein levels [64].

**Table 4:** microRNAs targeting FOXO Isoforms according to the DIANA-TarBase v8 (http://www.microrna.gr/tarbase) indexing experimentally supported miRNA–gene interactions [143].

FOXO1	FOXO3	FOXO4	FOXO6
hsa-let-7a-5p	ebv-miR-BART19-3p	hsa-miR-103a-3p	hsa-let-7b-5p
hsa-let-7b-5p	hsa-let-7f-2-3p	hsa-miR-1292-5p	hsa-miR-1343-3p
hsa-let-7c-5p	hsa-miR-10b-5p	hsa-miR-1304-5p	hsa-miR-146a-5p
hsa-let-7e-5p	hsa-miR-126-3p	hsa-miR-1-3p	hsa-miR-155-5p
hsa-let-7f-5p	hsa-miR-129-2-3p	hsa-miR-146a-5p	hsa-miR-16-5p
hsa-let-7g-5p	hsa-miR-141-3p	hsa-miR-1908-5p	hsa-miR-22-3p
hsa-let-7i-5p	hsa-miR-146a-5p	hsa-miR-21-3p	hsa-miR-7-5p
hsa-miR-10a-3p	hsa-miR-147a	hsa-miR-224-5p	
hsa-miR-10b-5p	hsa-miR-148b-3p	hsa-miR-27a-5p	
hsa-miR-133a-3p	hsa-miR-152-3p	hsa-miR-31-5p	
hsa-miR-141-3p	hsa-miR-155-5p	hsa-miR-3179	
hsa-miR-146a-5p	hsa-miR-155-5p	hsa-miR-335-5p	
hsa-miR-155-5p	hsa-miR-182-5p	hsa-miR-335-5p	
hsa-miR-15a-5p	hsa-miR-182-5p	hsa-miR-335-5p	
hsa-miR-16-5p	hsa-miR-185-3p	hsa-miR-335-5p	
hsa-miR-17-3p	hsa-miR-18a-3p	kshv-miR-K12-6-3p	
hsa-miR-17-5p	hsa-miR-19a-3p		
hsa-miR-181c-3p	hsa-miR-19b-3p		
hsa-miR-182-5p	hsa-miR-200a-3p		
hsa-miR-183-5p	hsa-miR-206		
hsa-miR-196a-5p	hsa-miR-21-3p		
hsa-miR-200b-3p	hsa-miR-21-5p		
hsa-miR-203a-3p	hsa-miR-218-5p		
hsa-miR-210-3p	hsa-miR-221-3p		
hsa-miR-221-5p	hsa-miR-222-3p		
hsa-miR-223-3p	hsa-miR-223-3p		
hsa-miR-27a-3p	hsa-miR-22-3p		
hsa-miR-324-3p	hsa-miR-23a-3p		
hsa-miR-324-5p	hsa-miR-23b-3p		
hsa-miR-330-3p	hsa-miR-26b-3p		
hsa-miR-335-5p	hsa-miR-27a-3p		
hsa-miR-33a-3p	hsa-miR-27b-3p		
hsa-miR-369-3p	hsa-miR-296-3p		
hsa-miR-374a-5p	hsa-miR-29a-3p		
hsa-miR-3934-5p	hsa-miR-29b-3p		
hsa-miR-424-5p	hsa-miR-29c-3p		
hsa-miR-452-5p	hsa-miR-3065-3p		
hsa-miR-4803	hsa-miR-335-5p		
hsa-miR-494-3p	hsa-miR-335-5p		
hsa-miR-499a-3p			
hsa-miR-501-3p			
hsa-miR-502-3p			
hsa-miR-503-5p			
hsa-miR-532-5p			
hsa-miR-544a			
hsa-miR-545-3p			
hsa-miR-548am-5p			
hsa-miR-548au-5p			
hsa-miR-548c-5p			
hsa-miR-548d-5p			
hsa-miR-548j-5p			
hsa-miR-548o-5p			
hsa-miR-582-3p			

Interestingly, FOXO proteins are involved in several feedback loops. FOXO factors can control their own expression adding a new level of complexity. FOXO1 can induce its expression, while FOXO3 can elevate FOXO1 and FOXO4 mRNA levels. The FOXO1 promoter exhibits a conserved FOXO-binding site involved in this positive feedback loop, whereas no conserved binding site is present in FOXO4 [53]. Furthermore, both FOXO1 and FOXO3 can induce AKT, their canonical inhibitor, via mTORC1 inhibition and mTORC2 activation, contributing to the maintenance of cellular energy homeostasis [65–67].

### Expression of FOXO isoforms

**Fig. 4:**
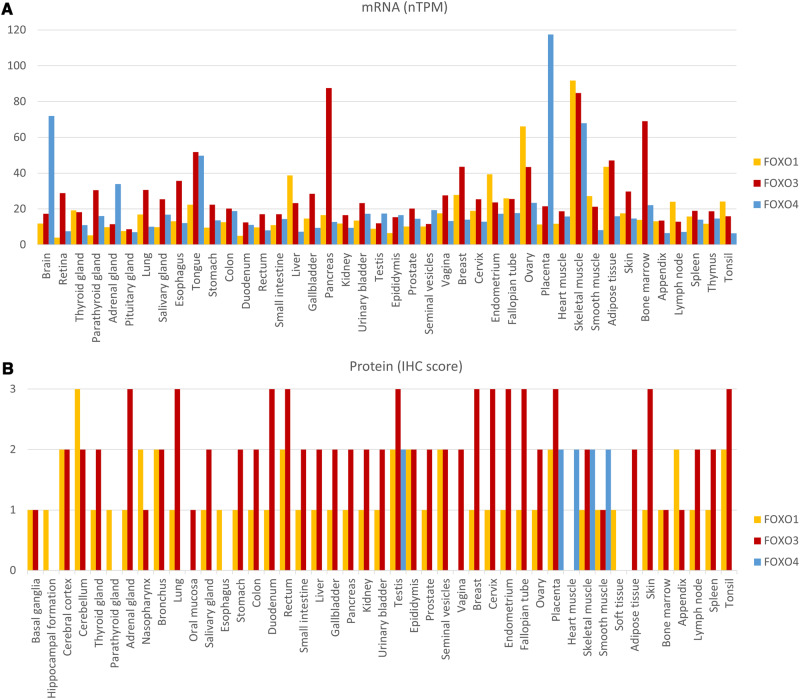
Expression of FOXO isoforms. **A** mRNA expression levels across different tissues. The mRNA expression levels in human tissues are based on RNA-seq data downloaded from Human Protein Atlas (HPA), which is a combination of the data from HPA and Genotype-Tissue Expression (GTEx) datasets. **B** FOXO family protein levels across different tissues. The protein levels in human tissues are based on immunohistochemistry data downloaded from Human Protein Atlas (HPA). Each bar represents the highest expression score found in a particular group of tissues.

As discussed above, the degree to which context-dependent mechanisms such as tissue-specific expression of FOXO isoforms contribute to their differential functions is still controversial. Regardless of the point of view, an accurate and comparative characterization of transcript and protein expression of each isoform in humans, rodents and other species represents an important piece of information to better understand differential isoform functions. According to Human Protein Atlas (HPA) (www.proteinatlas.org), human FOXO transcripts except FOXO6 mRNAs, are ubiquitously expressed, but at different levels depending on the tissue site (Fig. 4A). Some tissues exhibit considerably higher levels of overall FOXO transcripts than others, like the brain, pancreas, placenta, skeletal muscle, and bone marrow. Moreover, not all isoforms are expressed equally within each tissue. FOXO1 is the prevalent isoform in the thyroid, liver, endometrium, ovary, skeletal muscle, smooth muscle, lymph node, and tonsil. FOXO3 is the predominant isoform at the transcript level in the retina, parathyroid, lung, salivary, esophagus, tongue, stomach, colon, duodenum, rectum, small intestine, gallbladder, pancreas, kidney, bladder, prostate, vagina, breast, cervix, adipose tissue, skin, bone marrow, appendix, spleen, and thymus. FOXO4 is the most expressed FOXO transcript in the brain, adrenal gland, testis, epididymis, seminal vesicles, and placenta. FOXO6 mRNA is only detected, at very low levels, in the brain (data not shown).

Transcript levels might be useful to predict FOXO isoform expression but do not necessarily portray protein levels and, more importantly, their activity. Unfortunately, there is a lack of means to consistently and reliably detect and quantify the level of FOXO proteins in tissues. The biggest concern is related to the poor quality and/or low specificity of commercially available antibodies that can bias evaluation. While HPA data on protein levels (Fig. 4B), determined by immunohistochemistry (IHC), display low consistency between antibody staining and RNA expression for FOXO1, the data are more consistent for FOXO3 and FOXO4, yet discrepancies also exist for the latter isoforms. Tissue annotation of FOXO6 is still pending. Nonetheless, data indicate that FOXO3 protein levels prevail in the majority of tissues, while FOXO4 protein expression is dominant in the testis, placenta and heart, skeletal, and smooth muscles.

Another factor limiting the accurate expression analysis of FOXO isoforms is the lack of comparative studies. The number of studies that compare multiple isoforms simultaneously in human samples is still scarce. FOXO1, FOXO3, and FOXO4 transcripts are detected in thyroid samples, although their levels were not compared [68]. Likewise, in gestational tissues, FOXO1, FOXO3, and FOXO4 mRNA and proteins are detected in the placenta and fetal membranes [69]. FOXO1 and FOXO3 are the prevalent isoforms at mRNA level in mature endothelial cells, while FOXO4 is comparably low [70]. Another study reported the expression of FOXO1, FOXO3, and FOXO4 proteins in saphenous vein smooth muscle cells. The authors showed that FOXO1 and FOXO3, but not FOXO4 regulated the proliferation of these cells [71]. FOXO1, FOXO3, FOXO4, and FOXO6 are detected in gastric tissues [71, 72], and skeletal muscles [73], while only FOXO1, FOXO3, and FOXO4 have been reported in bone cells [74]. In the skin, although all isoforms can be found, only FOXO1 is associated with epidermal morphogenesis and FOXO3 with melanin biosynthesis suggesting a non-redundant function in these cells while the role of FOXO4 and FOXO6 remains to be explored [75]. FOXO1, FOXO3, and FOXO4 are also expressed in human embryonic stem cells, FOXO1 being the most abundant isoform. FOXO1 and FOXO3 are involved in pluripotency regulation under homeostatic or stress conditions, respectively, while FOXO4 is associated with neuronal lineage commitment [76].

FOXO6, the last family member to be discovered, is the least characterized isoform which makes it difficult to compare its expression with the others. FOXO6 expression was thought to be limited to the brain, with the highest levels found in the pituitary gland [22, 76]. Nevertheless, it is also expressed in peripheral tissues, including the lungs, liver, kidneys, intestine, bone, muscle, and adipose tissue [77–80].

In mice, FOXO1, FOXO3, and FOXO4 are present at high levels in insulin-responsive tissues, where FOXO1, FOXO3, and FOXO4 were more strongly expressed in adipose tissue, liver, and muscle, respectively [81]. Although FOXO1 seems to be the predominantly expressed isoform in mice adipose tissues, FOXO3 and FOXO4 are also found and contribute along with FOXO1, in regulating metabolic crosstalk with liver and pancreatic β cells [82]. In mice liver, apart from FOXO3, FOXO1 and FOXO4 can also be found. In this tissue, FOXO1 and FOXO3 tend to respond to hormones and nutritional status through post-translational modifications, while FOXO4 is regulated at the transcriptional level [83]. These three isoforms are also important for cartilage development and homeostasis [84], quiescence, and survival of lymphocytes [85], and are expressed in the rodent ovary at specific stages of follicular development and luteinization [86]. In the rat duodenum, FOXO4 and FOXO3 proteins are expressed in a cell-specific and age-dependent pattern, while FOXO1 is not detected [87].

FOXO expression has also been analyzed throughout development in other species, including in frogs [88, 89] and primates [88]. FOXOs distinct chromosomal location can be responsive by their differential expression since each isoform will depend on the specific chromatin environment, promoters, enhancers, and regulatory regions, to be expressed. Even though the FOXO family of proteins is ubiquitously expressed, each isoform exhibits different expression patterns that might be responsible for target specificity.

### Isoform-specific functions

Characterizing non-redundant functions of FOXO isoforms is challenging for several reasons. Manipulation of one FOXO isoform can affect the expression or the activity of other isoforms, because their expression and activation are not independent. The most prominent example is the observation that FOXO1 induces the expression of RICTOR [67], a component of the mTORC2 complex that is required to mediate AKT-dependent inactivation of FOXO3 [90]. In addition, FOXO3 can increase the transcript level of PIC3CA [91] and FOXO1 [68]. On the other hand, FOXO6 has been reported to repress the expression of FOXO3 [24]. As there is solid evidence that FOXO proteins affect the expression of their paralogs [92, 93], the expression of FOXO isoforms is autonomous, but not independent. It is also important to note that many phenotypes reported upon manipulation of one FOXO isoform are not necessarily isoform-specific as most of these studies do not cover all isoforms. The absence of FOXO inactivating mutations in cancer might reflect the difficulty to get rid of four partially redundant transcription factors. The observation that the simultaneous ablation of three FOXO isoforms in mice is required to produce a mild cancer phenotype also suggests redundant tumor suppressor functions of FOXO1, 3, and 4 [32]. However, the current animal models including FOXO1, 3, and 4 null animals do not truly represent a FOXO null background because of the existence of FOXO6. There is clear evidence for non-redundant functions of FOXO isoforms largely based on isoform-specific depletion in cellular and animal models. However, the resulting phenotype might reflect expression effects rather than intrinsic differences of the isoforms. According to this concept, the depletion of a single isoform would only affect those cells or tissues in which the other isoforms are not expressed and are unable to compensate. This could explain that the spectrum of cancer types in the triple FOXO1, 3, and 4 knockout mice are limited to a few lineages.

### FOXO isoforms and longevity

FOXOs have emerged as longevity genes based on evidence from animal models including Hydra, C elegans, Drosophila, and mice [36]. While FOXO3 has been associated with exceptional longevity in multiple human populations, FOXO1 was found to be associated with longevity traits in female Han Chinese populations [94, 95]. In contrast, in Germans FOXO1, 4 or 6 genes don’t seem to play a significant role in the ability to reach old age [96]. As Europeans and Chinese have different FOXO1 linkage disequilibrium structures, the association of FOXO1 with human longevity might represent a Chinese- or Asian-specific effect. Protective alleles of FOXO3 are the second most-replicated genetic factors found so far to be associated with long life in humans [97]. About 40 common, noncoding single nucleotide polymorphisms (SNPs) have been identified within the FOXO3 locus affecting human longevity. The mechanism by which these FOXO3 alleles confer longevity is not linked to known coding SNPs in an exon but are rather located in or near intron 2 including the rs2802292 polymorphism (Fig. 1) [98] and it remains to be established how these variants can affect longevity. The presence of SNPs might modify the binding affinity of transcription factor/enhancer to FOXO3 or in the proximity of FOXO3 [99] or disrupt splicing regulatory elements leading to alternative isoforms [26, 100]. Although the activity of FOXO transcription factors and their target genes are closely related to many hallmarks of aging [101], it is poorly understood which biological process regulated by FOXOs is the most predominant one for human longevity. It is reasonable to consider that one of several FOXO-mediated processes including oxidative stress resistance, stem cell maintenance, DNA damage response, metabolism or autophagy or their combination supports exceptional longevity in humans. From a conceptual point of view, it is possible that cellular resistance to environmental stressors mediated by FOXO3 most significantly affects stem cell homoeostasis counteracting the gradual loss of the ability of adult stem cell populations to regenerate tissues [102]. However, it is important to note that FOXO plays an important role in the lifespan of C elegans, which exists as an adult organism exclusively of post-mitotic cells and lacks adult stem cells and regenerative potential.

Only two of the three FOXO isoforms in C elegans, namely Daf-16a, Daf-16d/f/h, but not Daf-16b play a critical role in the extension of lifespan [27, 103] indicating an isoform-specific effect that might also be relevant for the FOXO paralogs in mammals. Interestingly, a recent study investigated the role of Forkhead box family transcription factors including the FOXO isoforms in mouse somatic cell reprogramming and reported that FoxO6 but not the other FOXO isoforms almost totally block reprogramming through the inhibition of cell proliferation, suppressing the expression of pluripotent genes and hindering the process of mesenchymal to epithelial transition [27, 103, 104].

Caloric restriction (CR) is the most reliable intervention to regulate aging and increase the healthy lifespan in diverse species, including rodents and non-human primates [105] and FOXO proteins are thought to play a critical role in mediating the effects of CR. Interestingly, upon depletion of FOXO3 and even in the heterozygous state, mice cannot benefit from the lifespan extension effects of CR [106], while the lifespan of heterozygotic FOXO1 knockout mice (FOXO1^−/−^ mice died around embryonic day 11) did not differ under conditions of caloric restriction compared to wild type controls [107]. Although the roles for FOXO4 and FOXO6 in the effects of CR have not been studied, these data suggest an isoform-specific effect of FOXO3. It is still elusive why FOXO3 is the isoform predominantly associated with human longevity and to which degree other isoforms might contribute to the phenotypic response. Somewhat counterintuitively, FOXO4 has been shown to be critical in maintaining the viability of senescent cells by binding to p53 and repressing the p53-mediated apoptosis [108]. Disruption of the FOXO1/p53 interaction by the senolytic peptide FOXO4-DRI promotes clearance of senescent cells which are known to accelerate aging and is capable of restoring fitness, fur density and renal function in fast aging mice. FOXO4-DRI contains a segment of FOXO4 that binds to p53 and is conserved in both humans and mice but different from FOXO1 and FOXO3.

As aging is not a positively selected process, evolutionary theories of aging have raised the question on natural selection of longevity genes. Indeed, FOXO factors serve as paradigmatic examples of different mechanisms underlying aging theories [109] including the disposable soma theory that states that allocation of resources for growth/reproduction jeopardizes somatic maintenance [110]. Within this concept FOXO factors antagonize classical growth factor signaling promoting repair processes. Conversely, the quasi-programmed aging theory that focuses on signaling pathways beneficial earlier in life but driving aging later, identifies FOXO factors as gerosuppressors antagonizing chronic hyper-activation of the nutrient-sensing TOR pathway known to accelerate aging [110]. Regardless of deterministic or stochastic concepts of aging, it is still poorly understood if protective variants have specifically evolved at the FOXO3 locus because of unique features of the FOXO3 isoform or because FOXO3 is predominantly expressed in relevant tissues. It has been suggested that aging research is entering a new era that has unique implications for human health span [111] and that the discovery of longevity genes and pathways could guide drug development efforts aimed at development of new therapies.

### FOXO isoforms and cancer

FOXO factors have been established as context-dependent tumor suppressors that are frequently inactivated in human cancers [112, 113]. However, alterations in FOXO genes, either by chromosomal translocations or somatic point mutations have only been reported in a reduced number of cancers. Acquired chromosomal translocations have been reported for FOXO1, 3, and 4 resulting in fusion proteins and a loss of function of the respective FOXO isoform [113]. Consistent with the notion that the PI3K/AKT pathway is the most commonly activated pathway in human cancers [114], AKT-mediated phosphorylation represents the primary mechanism of FOXO inactivation in cancer. As mentioned above, the threonine and the two serine residues modified by AKT-mediated phosphorylation are conserved between FOXO/daf-16a in C elegans and the mammalian paralogs FOXO1, 3, and 4. This hardwired regulatory mechanism together with the observation that only the simultaneous depletion of these three FOXO isoforms results in a cancer phenotype in mice [32, 113] clearly points to redundant tumor suppressor functions of these proteins in cancer. Of note, in combined FOXO1/3- knockout or FOXO1/4- knockout animals, tumor incidence was slightly increased [32]. Furthermore, the absence of mutations in FOXO genes in human tumors also supports the notion of isoform redundancy in cancer. Without positive selection after one or two hits, independent mutations in three FOXO genes have little probability to occur [23]. However, it has been shown that FOXO1 but not FOXO3 mediates the tumor-inhibiting effect of CR [106]. The antineoplastic effect of CR, as indicated by reduced incidence of tumors at death in the wild type mice under conditions of CR, was mostly abrogated in heterozygotic FOXO1 knockout mice, in which the FOXO1 mRNA level was reduced by 50%, or less. Whereas haploinsufficiency of FOXO1 diminishes the antineoplastic effect of CR, homozygous and heterozygous ablation of FOXO3 gene in mice had no effect on the prevalence of tumors under CR compared to wild type animals [106]. These data indicate that isoform-specific functions of FOXO paralogs can become apparent under stress conditions such as CR and suggest differential regulation of cancer and lifespan by CR via FOXO1 and FOXO3. As FOXO factors are thought to be most needed under harsh conditions to maintain homeostasis, the protective properties of the FOXO isoforms might only surface in the presence of the insult they are supposed to react to. As mentioned earlier the output of FOXO activity depends on the cellular context and tumor suppressor activity is not always their predominant activity. It is important to note that the capacity of FOXO proteins, in particular FOXO1 and 3 to promote stem cell maintenance might also be relevant for cancer stem cells (CSCs). Yu et al. reported that FOXO1 promotes CSCs in breast cancer, by enhancing transcriptional expression of SOX2, a master regulator of stemness, which in turn, activates FOXO1 transcription and forms a positive regulatory loop [115]. Similarly, FOXO3 depletion can induce differentiation of myeloid leukemia cells, reducing leukemic-cell growth [116, 117]. In pancreatic ductal adenocarcinoma FOXO3 is able to induce stem cell properties, associated with poor prognosis and possibly metastasis [118, 119]. Conversely, in glioblastomas, FOXO3 activation promotes differentiation of CSCs, suppressing their tumorigenicity [120], and in colorectal cancer, FOXO3 activation inhibits CSCs self-renewal by significantly inducing TRAIL and its receptor DR5 [121].

### FOXO isoforms in diabetes

As mentioned above, DAF-16/FOXO proteins are important mediators of insulin signaling, regulating a variety of metabolic functions in a broad range of organisms [122, 123].

Diabetes is a metabolic disease characterized by elevated levels of blood glucose caused by impaired capability to produce or respond to insulin. Since FOXO proteins are key mediators of insulin signaling, they have been suggested as potential therapeutic targets for the treatment of diabetes.

All four FOXO isoforms are negatively regulated by insulin and insulin like growth factor (IGF) [77, 80, 124]. While FOXO1, 3, and 4 are inactivated via their translocation from the nucleus to the cytoplasm after AKT phosphorylation, the FOXO6 isoform remains in the nucleus regardless of its phosphorylation state [77, 125]. However, it has been hypothesized that phosphorylation of the two AKT phosphorylation sites in FOXO6 precludes FOXO6 binding to target promoters [125]. Conversely, a study demonstrated that FOXO6 was unable to interact with CRM1, an exportin that binds to the NES motif of a protein and is responsible for its nuclear export. Accordingly, FOXO6 does not contain a NES motif and thereby fails being transported between the nucleus and the cytoplasm [80].

The FOXO1 isoform regulates hepatic glucose production by decreasing both glycogenolysis and gluconeogenesis. Decreased expression of hepatic FOXO1 is associated with lower levels of glucose in mice, both at birth and in adulthood. FOXO1 plays an important role in the induction of hepatic gene expression and on the levels of glucose production and the major differences when comparing with the control mice are observed in animals showing temporary and not chronically decreased levels of FOXO1. This indicates that FOXO1 is required for the control and promotion of the hepatic glucose production, even in the presence of FOXO3 isoform, being FOXO3 able to compensate for chronic but not acute FOXO1 ablation [126]. Moreover, a recent in vivo study in mice showed that hepatic FOXO activity was upregulated in injury-caused hyperglycemia, demonstrated by the increased transcriptional levels of several FOXO target genes including IGFBP1, G6PC, PCK1, GCK, and PGC1a. Hepatic loss of FOXO1 in these conditions was associated with lower levels of insulin, when compared with the controls, without significant changes in the levels of glucose, suggesting that mice lacking FOXO1 were more sensitive to insulin after experiencing stress. However, to understand if other FOXO isoforms were contributing to this outcome, under these specific stress conditions, the authors evaluated the combined loss of FOXO1, FOXO3, and FOXO4 [127]. In fact, in mice lacking these three FOXO isoforms in the liver, the levels of both insulin and glucose significantly decrease when compared to the wild type animals indicating a protective and synergistic effect of the depletion of other FOXO isoforms in stress-induced hyperglycemia [127–129]. A recent study showed that FOXO1 interacts with PPARα and attenuates PPARα-mediated induction of the fibroblast growth factor 21 (FGF21), a hormone known to exert potent anti-diabetic and lipid-lowering effects [130]. In a different study, hepatic FOXO1 ablation in mice was compared with the simultaneous knockout of FOXO1 and FOXO3 and the triple ablation of the three isoforms, FOXO1, FOXO3, and FOXO4 in the liver. In all of these conditions, the glucose levels were decreased when compared with the wild type control animals, with the lowest glucose levels being associated with the simultaneous deletion of the three isoforms. Furthermore, in mice lacking the three isoforms, an increased insulin sensitivity was observed, when compared with the ablation of only one or two FOXOs, and the controls [129]. This data suggests once again a synergistic role between FOXO isoforms and their contribution to hepatic glucose production and insulin resistance.

Similarly, FOXO6 is also involved in insulin signaling and glucose production. Increased FOXO6 activity in the liver is associated with insulin resistance and insulin deficiency in mice (in fasting hyperglycemia and diabetic mice). FOXO6 transcription is induced by glucagon via cAMP and inhibited by insulin signaling. The same study also demonstrated the association between Foxo6 expression and induced hepatic gluconeogenesis, without changes verified in the transcriptional levels of the other isoforms (FOXO1, 3, and 4) [80]. According to the different data available, insulin signaling takes two primary and parallel routes downstream of AKT, one by FOXO1 and another by FOXO6 phosphorylation. The authors of this study suggest that these two routes complement each other and compensate for the failure of the other to maintain a functional liver.

The relevance of FOXO3 and FOXO4 in hepatic glucose production is not very clear yet, however, they contribute and play a synergistic role with FOXO1 and FOXO6 in the regulation of this process [129]. In fact, pediatric patients with type 1 diabetes mellitus showed significant FOXO3 upregulation, when compared with the healthy controls, suggesting a role of FOXO3 in the development of this disease [131]. Moreover, overexpression of FOXO3 was recently shown to be correlated with the destruction of pancreatic islets, which proposes a mechanism through which FOXO3 could contribute to type 1 diabetes mellitus [131].

FOXO isoforms, in response to insulin signaling, are, therefore, important regulators of the hepatic glucose production and the pancreatic β-cells, which demonstrates the importance of these transcription factors in the regulation of insulin sensitivity and diabetes. FOXO1 is the most studied isoform in this disease, but there is also evidence for a role of FOXO3 and FOXO6. However, the manipulation of all the isoforms leads to similar results in this context. While basal levels of FOXOs expression are beneficial and necessary for the regulation of some hepatic processes, their upregulation is associated with pathological phenotypes and the worst prognosis.

### FOXO isoforms and neurodegenerative diseases

FOXO transcription factors have been shown to play a role in neural cell fate and function, in both physiological and pathological conditions. Neurodegenerative diseases, such as Huntington’s, Parkinson’s, and Alzheimer’s diseases are associated with impaired neuronal and motor function, impacting the individual’s life over the years [51, 132].

In aging brain tissues, FOXO1 expression and activation seem to be significantly increased when compared with younger tissue, which was not verified for FOXO3 and FOXO4. However, simultaneous deletion of Foxo1, 3, and 4 in neurons seemed to be sufficient for initiating axonal degeneration, suggesting an important role of FOXO proteins in neuronal protection. Intriguingly, specific genes found overexpressed in aged brains, are also upregulated in adult mice lacking Foxo1, 3, and 4 expression [133]. This data suggests that basal expression of FOXO isoforms plays a protective role in neural degeneration and that their deregulation relates with neuron degeneration.

In Huntington’s disease, FOXO3 is overexpressed and seems to regulate its own expression in a positive autoregulation loop. FOXO3 overactivity might trigger a protective response to the elevated cellular stress [93]. In mice with a mutated HTT gene, a characteristic of this disease, deficiency in XBP1 protein plays a protective role for neuronal survival that seems to be correlated with higher levels of autophagy. In fact, decreased XBP1 seems to increase FOXO1 accumulation and activity. FOXO1 ectopic expression was also able to induce autophagy in this model, suggesting that XBP1 downregulation plays a protective role through FOXO1 induced autophagy [134].

In dopaminergic neurons, FOXO3 activity is related with neuronal death and ROS detoxification in oxidative stress conditions. When α-synuclein is overexpressed in these neurons, which happens in Parkinson’s disease, moderate FOXO3 expression seems to regulate α-synuclein accumulation, possibly via autophagic degradation. In this condition, but with increased levels of FOXO3 it was observed an accumulation of α-synuclein aggregates, contributed to the opposite effect and promoted cell death [135]. Overall, the basal expression and activity of FOXO transcription factors in neuronal diseases is associated with a protective role, in response to the developed stress.

A recent study regarding FOXO3 expression revealed a cell- and age-dependent function of FOXO3 in the brain of mice. Loss of FOXO3 was associated with altered metabolism and function of astrocytes, increased β-amyloid plaques, a pathological hallmark of Alzheimer’s disease, and synapse loss. Overall, FOXO3 expression in astrocytes seems to protect the brain from aging and from Alzheimer’s disease [136].

Additionally, it was observed that FOXO1 and FOXO3 could be potential targets in anxiety and mood disorders. Treatments in mice with imipramine, an antidepressant compound, were correlated with increased levels of FOXO1 and FOXO3 phosphorylation and consequent inactivation. In the same way, decreased FOXO1 levels in the brain were associated with reduced levels of anxiety in mice, being suggested as a regulator of the anxiety-like phenotypes [137].

In conclusion, normal expression and activity of FOXO proteins, specially FOXO1 and FOXO3, have a protective role in neuronal tissue and consequently in preventing neurodegenerative diseases in mice.

## Conclusions and future pesrpectives

Non-ligand transcription factors including FOXO proteins have been considered difficult to target due to significant structural disorder and lack of defined small-molecule binding pockets [138]. While significant advances in drug development methods enable pharmacological approaches to modulate the functions of FOXO transcription factors for therapeutic purposes [139], the benefits of targeting FOXO proteins will depend on our understanding of the diversity of FOXO functions and the role of isoform-specific activities. Importantly, this also includes the knowledge about the role of the variants derived from single FOXO genes. Solid evidence has been uncovered to support the assertion that the four FOXO paralogs in mammals have both overlapping and non-redundant functions. The pivotal common denominator of their functionality is the restricted recognition of enhancer elements in the promoters of target genes by a highly conserved forkhead domain. Conversely, paralog-specific, non-redundant functions are fueled by a range of intrinsic and context-dependent sources including differences in the paralog genes, transcripts and proteins, their regulation by miRNAs, PTMs and binding partners as well as their cell-specific expression and regulation. However, the contribution of each of these mechanisms to non-overlapping functionalities is still poorly understood. It is important to point out that the possibility that most of the paralog-specific functions are merely a result of their tissue-specific expression and co-expression patterns cannot be ruled out. Our knowledge of paralog-specific functionalities is patchy as FOXO isoforms have mostly been studied individually. Very few studies have been conducted comparing the FOXO paralogs side by side. No systematic research covering all paralogs has been performed to analyze tissue-specific expression and co-expression, cell-specific regulation such as treatment-induced nuclear translocation or target gene profiles. Indeed, paralog co-expression profiling might uncover a rationale for tissue-specific functional redundancy as a potential failsafe mechanism for defense systems to maintain cellular homeostasis. Several scenarios for isoform-specific FOXO targeting seem plausible. In tissues in which only one FOXO isoform is expressed or dominant, its activation or inhibition is specific in the absence of compensatory mechanisms. Inhibition of an upstream regulatory protein that induces a FOXO isoform-specific PTM might be achieved by small molecule compounds. Similarly, therapeutic peptides, peptidomimetics or small molecule compounds could be used to disrupt the interaction of FOXO isoform-specific binding partners. The observation that a FOXO3a^pS253^ phosphopeptide displays a distinct mode of interaction with 14-3-3 compared to FOXO1^pS256^ [37] might allow for the rational design of isoform-specific FOXO/14-3-3 protein-protein interaction inhibitors. Specific inhibition of the interaction between FOXO4 and p53 by a peptide called DRI has been demonstrated [108]. Furthermore, small molecules were identified that affect the binding of FOXO1 to SIN3A [140]. Differential binding of compounds to the DBD of FOXO isoforms based on subtle differences between these domains and their interaction with the consensus DNA sites could also be exploited. The emerging transcription-independent functions of FOXO proteins known to be involved in autophagy, apoptosis, and DNA damage response might provide yet another angle to target FOXO isoforms, although the specificity of these mechanisms are poorly understood.

miRNAs that bind to stabilizing sequences in the 3′ end of FOXO isoforms either stabilizing them or targeting them for degradation could be another approach. The availability of isoform-specific binding partners or small molecules could enable targeted protein degradation using proteolysis targeting chimaeras (PROTACs). But, maybe the most important issue to be addressed by these future studies is the feasibility and therapeutic usefulness of isoform-specific FOXO modulating drugs.

### Supplementary information

Figure S1

legend Figure s1

## Data Availability

The current manuscript is not an original research article and, therefore, does not require a data availability statement. All data presented within this article are available through the referenced publications.
