# Correction: FOXO family isoforms

**DOI:** 10.1038/s41419-023-06328-4

**Published:** 2023-12-06

**Authors:** Bruno F. Santos, Inês Grenho, Paulo J. Martel, Bibiana I. Ferreira, Wolfgang Link

**Affiliations:** 1 Algarve Biomedical Center Research Institute-ABC-RI, University of Algarve, Campus de Gambelas, 8005-139 Faro, Portugal; 2 Algarve Biomedical Center (ABC), University of Algarve, Campus de Gambelas, 8005-139 Faro, Portugal; 3 Faculty of Medicine and Biomedical Sciences, University of Algarve, Campus de Gambelas, 8005-139 Faro, Portugal; 4 Centro Hospitalar Universitário do Algarve (CHUA). Rua Leão Penedo, 8000-386 Faro, Portugal; 5 Center for Health Technology and Services Research (CINTESIS)@RISE, University of Algarve, Campus de Gambelas, 8005-139 Faro, Portugal; 6 Instituto de Investigaciones Biomédicas “Alberto Sols” (CSIC-UAM). Arturo Duperier 4, 28029 Madrid, Spain

**Keywords:** Transcription, Oncogenesis

Correction to: *Cell Death & Disease* 10.1038/s41419-023-06177-1, published online 27 October 2023

In this article the funding note needs to be added:

“This work was supported by a grant from Spanish Ministerio de Ciencia e Innovación/Agencia Estatal de Investigación and the European Regional Development Fund (PID2022-136654OB-I00 financed by MCIN/AEI /10.13039/501100011033 / FEDER, UE).”

The original article has been corrected.

