# Supplementary material for: FOXO family isoforms
Source: Cell Death Dis. 2023 Oct 27;14(10):702. doi: 10.1038/s41419-023-06177-1 (PMC10611805; doi:10.1038/s41419-023-06177-1)
Supplement: Supplementary file 1 — Figure S1 [file 41419_2023_6177_MOESM1_ESM.pdf]

```
FOXO1_HUMAN
                  1           10        20        30        40        50        60
1 FOXO1_HUMAN  ........MAEAPQVVEIDPDFEPLPRPRSCTWPLPRPEFSQSNSATSSPAPSGSAAAN.....PDAAAGLPSAS
2 FOXO3_HUMAN  MAEAPASPAPLSPLEVELDPEFEPQSRPRSCTWPLQRPELQASPAKPSGETAADSMIPEEEDDEDDEDGGGRAGS
3 FOXO4_HUMAN  MDPGNENSATEAAAIIDLDPDFEPQSRPRSCTWPLPRPEIANQPSEPPEVEPDLGEKVH...............
4 FOXO6_HUMAN  ......MAAKLRAHQVDVDPDFAPQSRPRSCTWPLPQPDLAGDEDGALGAGVAE....................

FOXO1_HUMAN
                    70        80        90       100       110       120       130
1 FOXO1_HUMAN  AAAV....SADFMSNLSLLEESEDFPQAPGSVAAAVAAAAAAATGGLCGDFQGPEAGCLHPAPPQPPPPGPLSQ
2 FOXO3_HUMAN  AMAIGGGGGSGTLGSGLLLEDSARV.LAPGGQDPG...SGPATAAGGLSGG....TQALLQPQQPLPPP......
3 FOXO4_HUMAN  ...T..EGRS....EPILL..PSRLPEPAGGP..........................................
4 FOXO6_HUMAN  .............................GAE..............DCGPERRATAPAMAPAP...........

FOXO1_HUMAN                                  TT              H1          S1     H2       H4
                 140       150       160       170       180       190       200
1 FOXO1_HUMAN  HPPVPPAAAGPLAGQPRKSSSRRNAWGNLSYADLITKAIESSAEKRLTLSQIYEWMVKSVPYFKDKGDSNSSAG
2 FOXO3_HUMAN  ....QPGA.AGGSGQPRK.CSSRRNAWGNLSYADLITRAIESSPDKRLTLSQIYEWMVRCVPYFKDKGDSNSSAG
3 FOXO4_HUMAN  ....QPGILGA.VTGPRK.GGSRRNAWGNQSYAELISQAIESAPEKRLTLAQIYEWMVRTVPYFKDKGDSNSSAG
4 FOXO6_HUMAN  ......PLGAEVGPLRKAKSSRRNAWGNLSYADLITKAIESAPDKRLTLSQIYDWMVRYVPYFKDKGDSNSSAG

             H3                 S2          S3
FOXO1_HUMAN                          TTT         TT
             210       220       230       240       250       260       270       280
1 FOXO1_HUMAN  WKNSIRHNLSLHSKFIRVQNEGTGKSSWWMLNPEGGKSGKSPRRRAASMDNNSKFAKSRSRAAKKKASLQSGQE.
2 FOXO3_HUMAN  WKNSIRHNLSLHSRFMRVQNEGTGKSSWWIINPDGGKSGKAPRRRAASMDNSNKYTKSRGRAAKKKAALQTAPE.
3 FOXO4_HUMAN  WKNSIRHNLSLHSKFIKVHNEATGKSSWWMLNPEGGKSGKAPRRRAVSMDSSSKLLRGRSKAPKKKPSVLPAPPE
4 FOXO6_HUMAN  WKNSIRHNLSLHTRFIRVQNEGTGKSSWWMLNPEGGKTGKTPRRRAVSMDNGAKFLRIKGKASKKKQLQAPERSP

FOXO1_HUMAN
                          290       300       310       320       330       340
1 FOXO1_HUMAN  .....G.AGDSPGSQFSKWPASPGSHSNDDFDNWSTFRPRTSSNASTISGRLSPIMTEQDDLG..E..GDVHSMV
2 FOXO3_HUMAN  .....S.ADDSP.SQLSKWPGSPTSRSSDELDAWTDFRSRTNSNASTVSGRLSPIMASTELDEVQDDDAPLSPML
3 FOXO4_HUMAN  .....GATPTSPVGHFAKWSGSPCSRNREEADMWTTFRPRSSSNASSVSTRLSPLRPESEVLAEEI...PASVSS
4 FOXO6_HUMAN  DDSSPSAPAPGPVPAAAKWAASPASHASDDYEAWADFRGGGRPLL....GE....AAELEDDEALEALAPSSPLM

FOXO1_HUMAN
             350            360                 370       380       390       400
1 FOXO1_HUMAN  YPP.SAAK.......MASTLPSLSEISNPE..........NMENLLDNLNLLSSPTSLTVSTQSSPGTMMQQTPCY
2 FOXO3_HUMAN  YSS.SASLSPSVSKPCTVELPRLTDMAGTMNLNDGLTENLMDDLLDNIT.......LPPSQPSPTGGLMQRSSSF
3 FOXO4_HUMAN  YAG.G.............V.........PPTLNEG.....LELLDGLNLTSSHSL..........LSRSGLS
4 FOXO6_HUMAN  YPSPASALSPALGSRCPGELPRLAELGGPLGLHGGGAGLPEGLLDGAQDAYGPRPAP.....RPG.........

FOXO1_HUMAN
               410       420       430       440       450       460       470
1 FOXO1_HUMAN  SFAPPNTSLNSPSPNYQKYTYGQSSMSPLPQMPIQTLQDNKS.SYGGMSQYNCAPGLLKELLTSDSP.PHNDIMT
2 FOXO3_HUMAN  PYTTKGSGLGSPTSSFNSTVFGPSSLNSLRQSPMQTIQENKPATFSSMSHYG..NQTLQDLLTSDSL.SHSDVMM
3 FOXO4_HUMAN  GFSLQHPGVTGPLHTYSSSLFSPAEG......PLS..........AGEGCF.SSSQALEALLTSDTPPPPADVLM
4 FOXO6_HUMAN  .......PVLGAPGE...................LALAGAAAAYPGKGAAPY.............APPAPSRS.

FOXO1_HUMAN
            480       490       500       510       520       530       540
1 FOXO1_HUMAN  PV.DPGVAQPNSRVLG....QNVMMGPNSVMSTYGSQASHNKMMNPSSHTHPGHAQQTSAVNGRPLPHTVSTMPH
2 FOXO3_HUMAN  TQSDPLMSQASTAVSAQNSRRNVMLRND.PMMSFAAQPNQGSLVNQNLLHH.QHQTQGALGGSRALSNSVSNMGL
3 FOXO4_HUMAN  TQVDPILSQAPTLLLLGGLPSSSKLAT............GVGLCPKPLEAPGPS.........SLVPTLSMI..
4 FOXO6_HUMAN  .....ALAHPI..................SLMTLPGEAG......AAGLAPPGHAAAF...GG.............

FOXO1_HUMAN
            550       560       570       580       590       600       610       620
1 FOXO1_HUMAN  TSGMNRLTQVKTPVQVPLPHPMQMS..ALGGYSSVSSCNGYGRMGLLHQEKLPSDLD.GMFIERLDCDMESIIRN
2 FOXO3_HUMAN  SE.SSSLGSAKHQQQSPVSQSMQTLSDSLSGSS...LYSTSANLPVMGHEKFPSDLDLDMFNGSLECDMESIIRS
3 FOXO4_HUMAN  .......APPPVMASAPIPKA.......LGTP......VLTPPTEAASQDRMPQDLDLDMYMENLECDMDNIISD
4 FOXO6_HUMAN  ..............P.PGGLLLDALPGPYA...AAAAGPL.GAAPDRFPADLDLDMFSGSLECDVESIILN

FOXO1_HUMAN
                    630       640                650
1 FOXO1_HUMAN  DLMDGDTLDFNFDNVLPNQS........FPHSVKTTTHSWVSG
2 FOXO3_HUMAN  ELMDADGLDFNFDSLISTQNVVGLNVGNFTGAKQASSQSWVPG
3 FOXO4_HUMAN  LMDEGEGLDFNFEPDP...........................
4 FOXO6_HUMAN  DFMDSDEMDFNFDSALPPPPPG.......LAGAPPPNQSWVPG
```
