# Supplementary material for: FOXO family isoforms
Source: Cell Death Dis. 2023 Oct 27;14(10):702. doi: 10.1038/s41419-023-06177-1 (PMC10611805; doi:10.1038/s41419-023-06177-1)
Supplement: Supplementary file 2 — legend Figure s1 [file 41419_2023_6177_MOESM2_ESM.docx]

**Suppl. Figure 1. Full-length alignment of FOXO isoforms.**

Red boxes pinpoint regions that exhibit complete conservation among all isoforms. The structurally conserved forkhead domain, essential for FOXO function, is outlined in green. Secondary structure information is provided, referencing the well-characterized FOXO1 structure. The three conserved regions (CR1, CR2, and CR3) are enclosed within distinct orange boxes. Areas of high sequence similarity are demarcated by blue outlines.
